# Toward community predictions: Multi‐scale modelling of mountain breeding birds' habitat suitability, landscape preferences, and environmental drivers

**DOI:** 10.1002/ece3.6295

**Published:** 2020-04-29

**Authors:** Nasrin Amini Tehrani, Babak Naimi, Michel Jaboyedoff

**Affiliations:** ^1^ Institute of Earth Sciences University of Lausanne 1015 Lausanne Switzerland; ^2^ Department of Geosciences and Geography University of Helsinki Helsinki Finland

**Keywords:** birds, habitat suitability model, multi‐scale model, presence‐only model, spatial scale, the western Swiss Alps

## Abstract

Across a large mountain area of the western Swiss Alps, we used occurrence data (presence‐only points) of bird species to find suitable modelling solutions and build reliable distribution maps to deal with biodiversity and conservation necessities of bird species at finer scales. We have performed a multi‐scale method of modelling, which uses distance, climatic, and focal variables at different scales (neighboring window sizes), to estimate the efficient scale of each environmental predictor and enhance our knowledge on how birds interact with their complex environment. To identify the best radius for each focal variable and the most efficient impact scale of each predictor, we have fitted univariate models per species. In the last step, the final set of variables were subsequently employed to build ensemble of small models (ESMs) at a fine spatial resolution of 100 m and generate species distribution maps as tools of conservation. We could build useful habitat suitability models for the three groups of species in the national red list. Our results indicate that, in general, the most important variables were in the group of bioclimatic variables including “Bio11” (Mean Temperature of Coldest Quarter), and “Bio 4” (Temperature Seasonality), then in the focal variables including “Forest”, “Orchard”, and “Agriculture area” as potential foraging, feeding and nesting sites. Our distribution maps are useful for identifying the most threatened species and their habitat and also for improving conservation effort to locate bird hotspots. It is a powerful strategy to improve the ecological understanding of the distribution of bird species in a dynamic heterogeneous environment.

## Graphical abstract

To know better how bird species and their complex environment interact, we have performed a multi‐scale modelling method using focal variables in different scales to evaluate the efficient scale of each environmental predictor where they are influential.

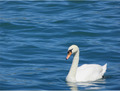

## 1 INTRODUCTION

Species populations are now more isolated and declining as a consequence of climate change and fragmentation and degradation of their habitats which also lead to erosion of mass biodiversity (Bosso et al., 2018; Haddad et al., 2015; Lindenmayer, Franklin, & Fischer, 2006; Zambrano et al., 2019). The influences of habitat spatial and ecological quality and alteration on species loss, isolation, recolonization, and movement have been widely acknowledged (e.g. Andrén, 1994; Collinge, 1998; Imbeau, Drapeau, & Mönkkönen, 2003; Wiens, 2002a, 2002b; Zambrano et al., 2019). In this context, landscape compositions and arrangements of natural areas, such as mountains, and knowledge of the interaction between species and their environment play a key role in population persistence and biodiversity conservation (Austin, 2002; Guisan & Thuiller, 2005; Scridel et al., 2018; Zambrano et al., 2019).

This could be achieved with modelling tools, such as species distribution models (SDMs), that facilitate conservation decisions in spatially complex landscapes (Guisan & Thuiller, 2005; Guisan et al., 2013; Habibzadeh & Ludwig, 2019). SDMs are statistical methods for assessing species' ecological requirements and predicting geographical distribution in space and time on the basis of the correlations of environmental variables and species occurrences (Austin, 2007; Hirzel, Lay, Helfer, Randin, & Guisan, 2006; Naimi & Araújo, 2016). When using SDMs, it is critical and problematic to identify the appropriate scale within which environmental variables affect the distribution of species and most SDMs fall short to incorporate environmental and land use variables at different spatial scales (Fournier, Barbet‐Massin, Rome, & Courchamp, 2017; Guisan & Thuiller, 2005; Mackey & Lindenmayer, 2001; Vicente et al., 2014). Most SDM studies rarely support the role of the best scale (the optimal range of spatial scales affecting distribution patterns of mobile organisms) of effects of focal predictors (that sum up information on the surrounding landscape within the focal cell; see Bellamy, Scott, & Altringham, 2013; Fournier et al., 2017; Guisan & Thuiller, 2005) on highly mobile organisms like bird species in complex landscapes (Chase et al., 2018; Guisan & Thuiller, 2005). SDMs usually consider a single predictor at a single spatial scale that is restricted to the extent of the sampling and the grain size and does not sufficiently address scale dependencies (Chase et al., 2018; Fournier et al., 2017; Hamer & Hill, 2000; Vicente et al., 2014). But the specific value of a single cell is not sufficient where we need several landscape elements, such as distance to resources, land structures, and land cover variables to incorporate spatial context and neighborhood and address the complexity of the ecological niche of the mobile species (Fournier et al., 2017; Guisan & Thuiller, 2005; Jaberg & Guisan, 2001; Rainho & Palmeirim, 2011; Scherrer, Christe, & Guisan, 2019).

Compared to plants, bird species pose more modelling problems at high spatial resolutions since it demands to take into account the effect of different spatial scales on a highly mobile group such as birds (Fournier et al., 2017; Guisan & Thuiller, 2005; Scherrer et al., 2019). In addition, research into feeding and nesting requirements for many bird species at multiple spatial scales still remains inadequate, and most of the recent studies tend to use a fixed spatial scale (Fournier et al., 2017; Jaberg & Guisan, 2001; Scherrer et al., 2019). Thus, it is important to develop an efficient framework, for bird species distribution, to assess the effect of focal predictors and compile information on the neighboring landscape within a focal cell at an appropriate scale (e.g., habitat selection in a proper scale; Bellamy et al., 2013; Bellier, Certain, Planque, Monestiez, & Bretagnolle, 2010; Bucklin et al., 2015; Fournier et al., 2017; Guisan & Thuiller, 2005; Revermann, Schmid, Zbinden, Spaar, & Schröder, 2012). Such a multi‐scale modelling method provides information on species distribution, identifies different requirements of bird species within foraging, nesting, and breeding range (Fournier et al., 2017; Guisan & Thuiller, 2005; Pearson, Dawson, & Liu, 2004; Vicente et al., 2014), and contributes to finer‐scale identification and planning of biodiversity and protected areas (Fournier et al., 2017; Razgour, Hanmer, & Jones, 2011; Vicente et al., 2014).

One major step forward of this study is the systematic assessment of the best scale within which the landscape (mainly land cover and land use), distance, topographic, and climatic predictors of bird habitat suitability are the most influential. This multi‐scale approach which involves linking focal variables with predicting power of ensembles of small models (ESMs) enables us to consider complexities in localization of birds as highly mobile species, especially in mountainous ecosystems with a great topographic ruggedness, which poses the significant modeling challenge of neighborhood analysis (Bellamy et al., 2013; Scherrer et al., 2019). These developments on the spatial modelling should improve our capacity to use models to assist bird monitoring and management in space and time, and ultimately help to identify priority bird conservation areas.

In this study, we employed the occurrence data of bird species to find efficient modelling solutions and build reliable distribution maps for biodiversity and conservation necessities at finer scales. In order to identify the efficient scale of each environmental predictor, we have performed a multi‐scale modelling method and used focal variables at different scales. We defined 12 neighboring window sizes (0.1‐5 km) to explore the effect of species‐specific scales of influence of each variable, and then we fitted univariate models to identify the best radius for each focal variable (land cover and land use) for each species and the most efficient scale of influence of each predictor. We then employed the final set of variables obtained from the previous stage to build ESMs at a fine spatial resolution of 100 m and generate species distribution maps as a monitoring tool of habitat suitability.

**FIGURE 1:**
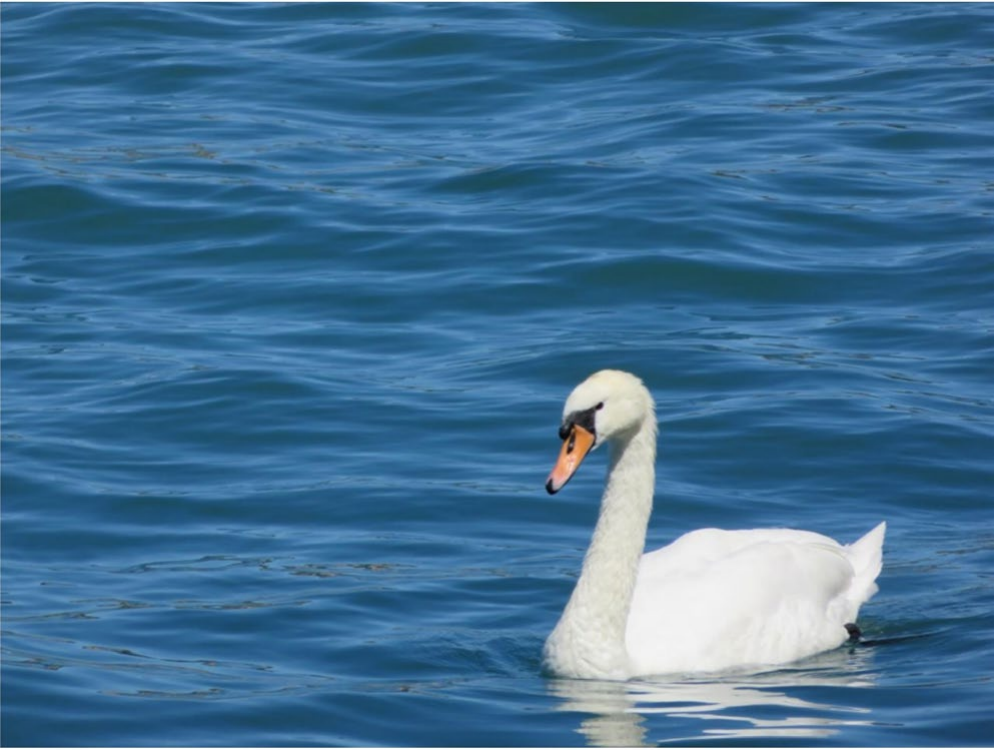
The Mute Swan (Cygnus olor) is an introduced breeding bird to Switzerland (Sattler et al., 2015)

The key issues in this research are the following: (a) an analysis of the role of the best scale (size of neighborhood) of effects of focal predictors (land cover and land use) at local (topographic and micro‐climatic) scale in bird species distribution across a complex landscape, and (b) providing potential and optimized distribution maps as a monitoring tool of habitat suitability and biodiversity conservation planning. This study followed a similar approach, carried out by Scherrer et al. (2019) to bat species, but we have expanded its capacity to include a diverse group of species (not just rare and cryptic species) and take into account the consequences of climate change and major anthropogenic disruptions in the landscape on bird species distribution for biodiversity planning purposes (Figure 1).

## 2 MATERIALS AND METHODS

### 2.1 Study area and species data

**FIGURE 2:**
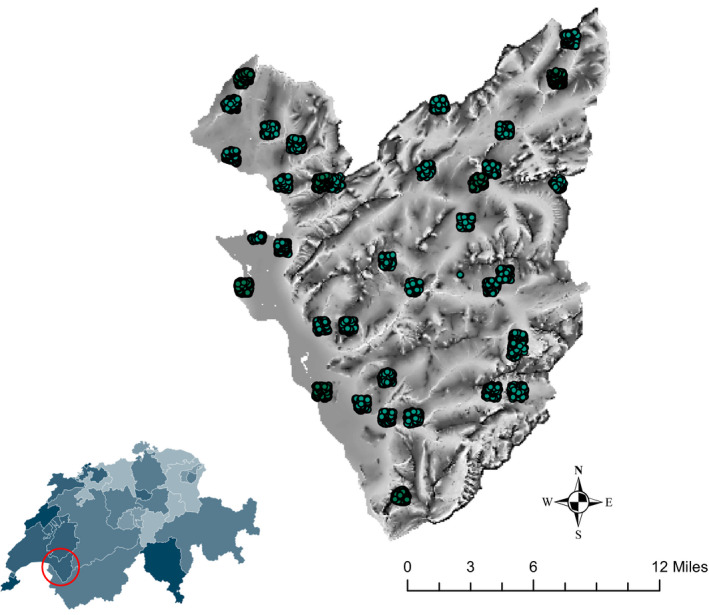
Study area and sampling points

The study area, the western Swiss Alps of Vaud (46°10 to 46°30′N; 6°50′ to 7°10′E; Figure 2), has been a site of high importance for interdisciplinary and transdisciplinary research at the University of Lausanne since 2013 and is now transferred to the Interdisciplinary Center for Mountain Studies (CIRM; http://rechalp.unil.ch). It includes an area of ca. 700 km^2^ and elevation gradient extending from Geneva's lake at 372 m to the Pointe des Diablerets at 3,210 m a.s.l. (Descombes, Vittoz, Guisan, & Pellissier, 2017; Scherrer et al., 2019) and it is affected by significant human activity: dense population and intensive farming in the Rhône Valley, tourism and leisure activities and more extensive farming in subalpine regions that makes up a mosaic of meadows, pastures, and forest and woodland patches (see http://rechalp.unil.ch; Pellissier et al., 2012; Randin, Jaccard, Vittoz, Yoccoz, & Guisan, 2009; Scherrer et al., 2019).

**FIGURE 3:**
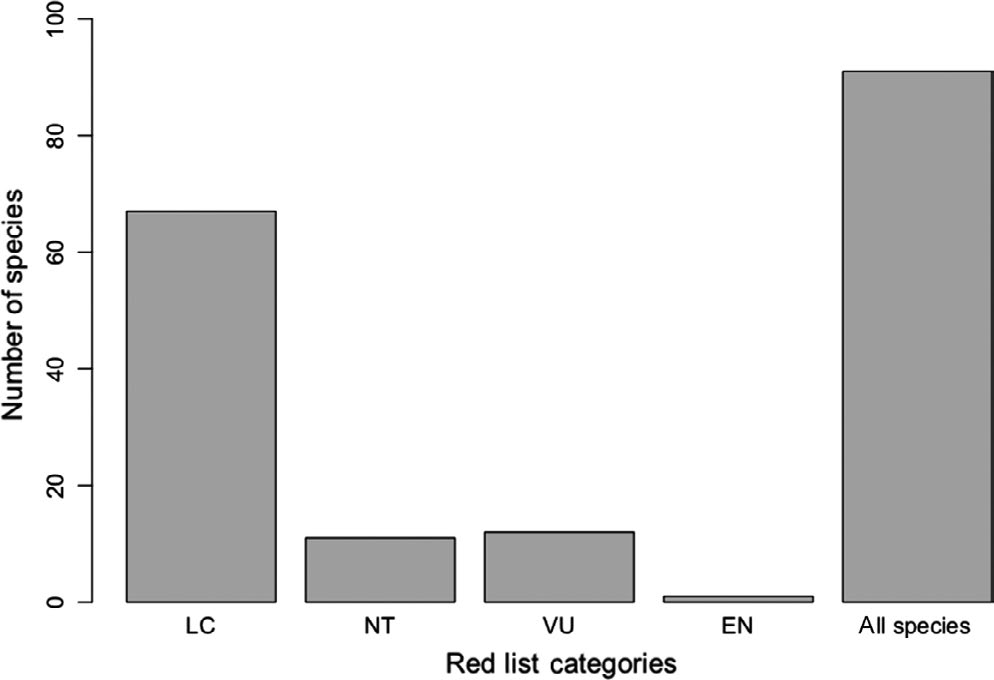
The number of species in each category of the Red List status. Species that were threatened, least concerned (LC), near threatened (NT), and vulnerable (VU)

The bird data are provided by the Swiss Ornithological Institute (Monitoring Häufige Brutvögel [MHB]; Schmid, Zbinden, & Keller, 2004) started in 1999 and performed annually (for more information on the survey see https://www.vogelwarte.ch/de/projekte/monitoring/monitoring‐haeufige‐brutvoegel), Swiss Breeding Bird Atlas 2013–2016 (Knaus et al., 2018; see https://www.vogelwarte.ch/en/projects/monitoring/swiss‐breeding‐bird‐atlas), and Swiss Biodiversity Monitoring [BDM] an ongoing biodiversity monitoring programme including birds species richness since 2001 that are updated annually, for monitoring the population of the most common species in terms of trends and changes of range size. Surveys and monitoring methods use those of the Sempach's Common Breeding Bird Survey (MHB) of the Swiss Ornithological Institute (BDM Coordination Office, 2014) that are explained in details in the following paragraphs (more details on http://www.biodiversitymonitoring.ch/en/background.html). These data are obtained from a systematic sampling (27,961 sampling points) of 267 quadrats, each covers 1‐km^2^, located as grids across Switzerland (39 quadrats are located in our study area). The data are collected three times in breeding seasons (15 April–15 July), and twice for quadrats that are located above the timberline at the elevation around 2,000 m. Each survey takes 3–4 hr along a 4‐6 km transect route where breeding birds are marked through visual observations or acoustic contacting (Kéry & Royle, 2009; Royle, Kéry, Gautier, & Schmid, 2007). A set of 91 bird species are used in this study with 67 species known as least concerned (LC), 11 species as near threatened (NT), 12 species as vulnerable (VU), and 1 as endangered (EN) based on the national red list (Figure 3). This classification could allow us to apply the research approach to a wide range of bird species from rare to poorly sampled ones. We only considered birds with a sample size greater than 20 presence records (>20 presence) as species with fewer presence records, due to errors connected with very small sample size, are not deemed appropriate for modeling (Thuiller, Lavorel, & Araújo, 2005).

### 2.2 Environmental data

Environmental data were derived from different sources, used for generating gridded environmental variables at 100 m spatial resolution and manipulated in ARCGIS 10.2 (Environmental System Research Institute, Inc.) or in R 3.3 (R Core team, 2016). We selected 15 environmental variables from various sources (Table 1) assumed ecologically meaningful and influential on mobile species like birds (Jaberg & Guisan, 2001). These include two climatic variables (Bio4 = Temperature Seasonality and Bio11 = Mean Temperature of Coldest Quarter), one topographic (slope), four distance variables (Euclidean distance to nearest lake, river, road, and residential area), the normalized difference vegetation index (NDVI; Rouse, Haas, Schell, & Deering, 1973), and canopy height mean. In addition, we defined a classification of six land cover and NDVI, each class measured within each pixel with varying 12 size (scales), i.e. within 12 neighboring windows of increasing radius size around each pixel (100, 200, 300, 400, 500, 1,000, 1,500, 2,000, 2,500, 3,000, 4,000 and 5,000 m; i.e. focal analyses—hereafter ‘focal variables’; Progin, 2018, Scherrer et al., 2019). Correlation coefficients were calculated for environmental variables, with the application of the Spearman's correlation coefficients (Spearman correlation <0.7; Dormann et al., 2013), to include the variables that are not highly correlated (Bellamy et al., 2013; Progin, 2018; Scherrer et al., 2019). There are more details on environmental variables in Table 1 and Table S1.

**TABLE 1:** Environmental variables. Description and name of each environmental variable used in the modelling process. The data were either provided by the OFS (Federal Office of Statistics) or OFT (Federal Office of Transports). For a more detailed description of the variables, please refer to Supporting Information

Category	Name	Description—Each layer is at a 100 M resolution	Source
Climatic	Bio 4	Temperature seasonality	Swisstopo OFT
	Bio 11	Mean temperature of Coldest Quarter	Swisstopo OFT
Topographic	Slope	Slope inferred from a digital elevation model at a 25 m resolution. Aggregate to 100 m resolution	Swisstopo OFT
Euclidean distance	Distoroad	Euclidean distance between the closet road and the center of the cell	Vector25 OFT
	Distozonhabit	Euclidean distance between the closet residential area and the center of the cell	Vector25 OFT
	Distolake	Euclidean distance between the closet lake and the center of the cell	Vector25 OFT
	Disriver	Euclidean distance between the closet river and the center of the cell	Vector25 OFT
Others	Canoheight_med	Average of canopy height at a 100 m resolution, calculating from a 1 m resolution raster	Swisstopo OFT
	NDVI_focal	Mean of normalized difference vegetation index at different focal scale	Swisstopo OFT
Land cover	Agriculture area	Proportion of agriculture area at different focal scale	Geostat OFS
	Forest	Proportion of forest at different focal scale	Geostat OFS
	Hedge	Proportion of hedge at different focal scale	Geostat OFS
	LAKE	Proportion of lake at different focal scale	Geostat OFS
	Orchard	Proportion of orchard at different focal scale	Geostat OFS
	Swamp	Proportion of swamp at different focal scale	Geostat OFS

### 2.3 Species distribution modelling

#### 2.3.1 Univariate models

**FIGURE 4:**
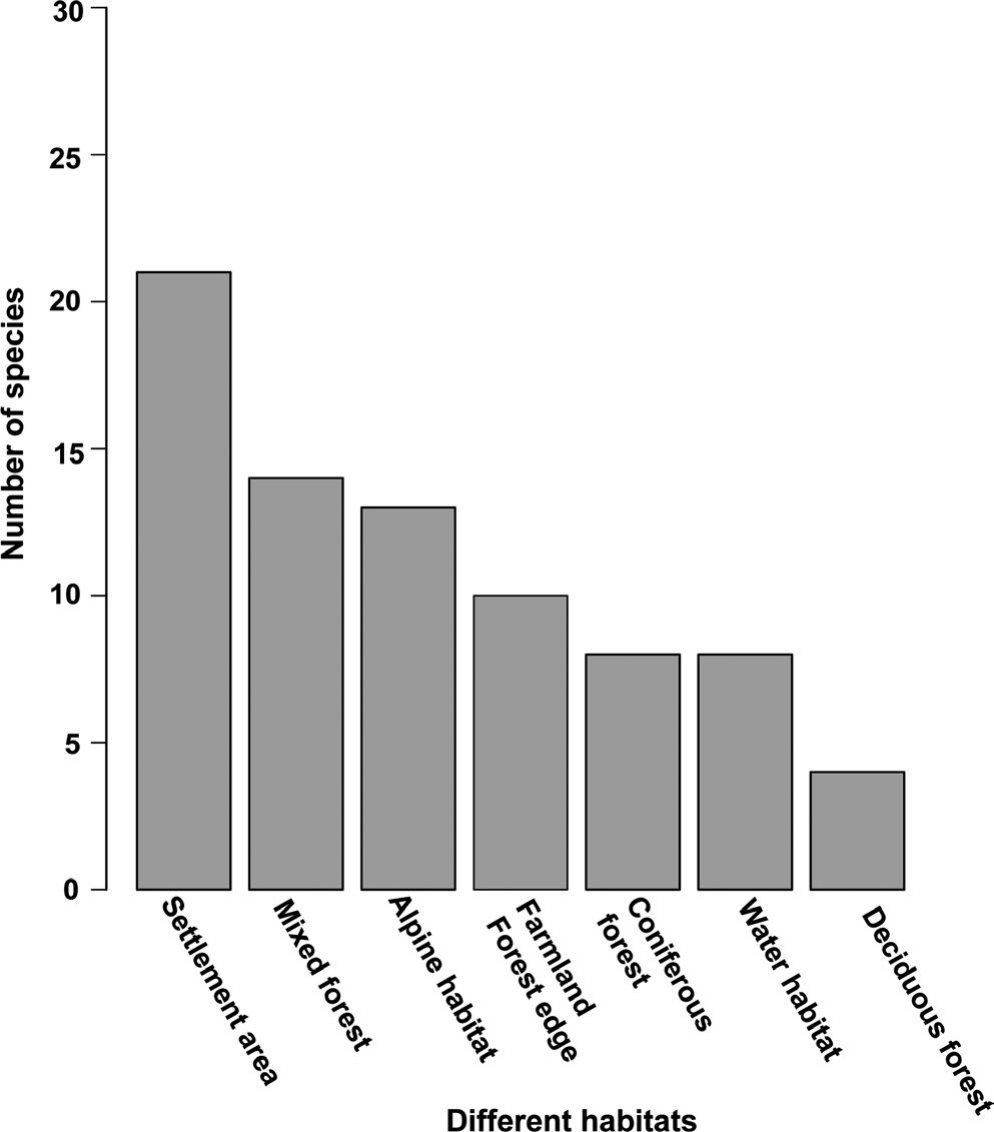
The number of bird species in different habitat types

For improving our understanding of the ecological connection between bird species and their complex environment, all bird species were grouped, based on their preferred habitat and settlements areas, into 7 groups including Alpine Habitat, Farmland and Forest Edge, Deciduous forest, Coniferous forest, Mixed Forest, and Water habitats (wetlands, lakes, rivers and streams; Bellamy et al., 2013; Herzog et al., 2005; Razgour et al., 2011; Figure 4).

For each species, we fitted univariate SDMs (Bellamy et al., 2013; Graf, Bollmann, Suter, & Bugmann, 2005; Progin, 2018; Scherrer et al., 2019) using a generalized linear model (GLM) technique to identify the best radius (size of the moving windows) for each focal variable. We used the biomod2 package to fit the models (Breiner, Guisan, Bergamini, & Nobis, 2015; Guisan, Edwards, & Hastie, 2002; Thuiller et al., 2016) in R v3.3 (R Core Team, 2016). For each focal variable, we run univariate models at the 12 neighboring window sizes (i.e. radius). Each model was run 10 times with 5 replicates of pseudo‐absence records with the size of 5,000, 50 runs in total, and at each run, 70% of the records were randomly selected to train the model, and the remaining 30% were used to evaluate the models. We used the area under the curve (AUC) of the receiver operating characteristic (ROC) plot (Fielding & Bell, 1997; Scherrer et al., 2019), which is the most frequently used metric in SDMs, to evaluate the models (Katz & Zellmer, 2018; Mohammadi, Ebrahimi, Moghadam, & Bosso, 2019; Scherrer et al., 2019). The pseudo‐absences were weighted equally to the presence (prevalence of 0.5; Ferrier, Drielsma, Manion, & Watson, 2002; Progin, 2018; Scherrer et al., 2019; Thuiller et al., 2016) as unbalanced prevalence reduce the accuracy of the models (Guisan, Thuiller, & Zimmermann, 2017). For each species, the best window radius size (i.e. scale) for each land‐cover variable was selected using the performance of the GLMs, measured by AUC, that was averaged for a better presentation of all bird data across all species, and run in different habitats. We selected the best scale (i.e. window size) for focal variables based on the highest AUC score, and all the variables with the highest AUC were selected and used later in the final model (multivariate models).

#### 2.3.2 Ensemble of small models

The ESMs was built in the "ecospat" package (Di Cola et al., 2017) and "biomod2" (Thuiller et al., 2016) in R 3.3 software (R Core Team, 2016) out of a large series of variables with just two variables as predictors (ESM; Breiner et al., 2015; Breiner, Nobis, Bergamini, & Guisan, 2018; Habibzadeh & Ludwig, 2019). We have used the birds’ occurrence data (presence‐only points) as the response variable and the final set of selected variables per species obtained from univariate models (previous stage) as predictors.

Two widely used modelling techniques, Generalized Additive Model (GAM) and Multiple Adaptive Regression Splines (MARS), showed powerful performance in fitting nonlinear and complex relationships between species (Leathwick, Rowe, Richardson, Elith, & Hastie, 2005; Mateo, Felicísimo, & Muñoz, 2010). All models were calibrated with presence only data combined with 10,000 randomly selected pseudo‐absence records (Breiner et al., 2015; Thuiller et al., 2016; Wisz & Guisan, 2009). Pseudo‐absence records were weighted to ensure an overall prevalence of 0.5 (i.e. giving equal weight to the presences and pseudo‐absences records (Progin, 2018; Scherrer et al., 2019; Thuiller et al., 2016). For each species, this process was repeated 10 times with each of 10 pseudo‐absence sets, 70% of the data were used for training and the other 30% for evaluating models (Araújo, Pearson, Thuiller, & Erhard, 2005; Muñoz & Felicísimo, 2004) with different evaluation metrics. Over the last years, there have been remarkable discussions about finding the best indices for evaluating models. Therefore, we used several indices such as area under the receiver operating characteristic curve (AUC), that is commonly used but recently criticized (Fernandes, Scherrer, & Guisan, 2019; Jiménez‐Valverde, 2012), the true skills statistic (TSS; Allouche, Tsoar, & Kadmon, 2006; Fernandes et al., 2019), Cohen's Kappa Statistic (KAPPA; Cohen, 1960; Fernandes et al., 2019), continues Boyce Index (CBI) recently developed as the most reliable metric for evaluating presence only data (Hirzel et al., 2006; Progin, 2018), and Somers'D metric (a re‐scaled version of the AUC that ranged between −1 and +1 (Breiner et al., 2015; Fernandes et al., 2019).

A fundamental tenet of ESMs, for each species, is to build bivariate small models with all possible combinations of two environmental variables as predictors that results in 105 models defined by (*n* ^2^ − n)/2, where n refers to the number of variables (Progin, 2018; Scherrer et al., 2019). Each of the 105 small bivariate models were weighted by Somers'D as a performing parameter D = 2 × (AUC − 0.5) that varies between −1 and +1 and gives higher weight to models that perform well (Breiner et al., 2015, 2018; Breiner, Guisan, Nobis, & Bergamini, 2017; Fernandes et al., 2019; Habibzadeh & Ludwig, 2019; Nielsen, Johnson, Heard, & Boyce, 2005; Progin, 2018; Scherrer et al., 2019). Then an ensemble forecast (Araújo & New, 2007) was generated by combining and averaging all small models for each modelling technique (ESM_GAM,_ ESM_MARS_; Habibzadeh & Ludwig, 2019; Progin, 2018; Scherrer et al., 2019). Finally, an ensemble prediction (ESM_EP_) was created by averaging the outputs of both the single model ESMs (ESM_GAM,_ ESM_MARS;_ Breiner et al., 2015). For each environmental variable contributing to ESMs (predicted by different bivariate models), the weighted mean was calculated using the mean weight of all bivariate models, variable of interest as well, regularized by the variable with the highest weight ranging between 0 and 1 (Razgour et al., 2011; Scherrer et al., 2019). This allows using importance value for each variable in the ESMs.

#### 2.3.3 Species distribution and richness maps

To create distribution maps, for three groups of species (all species, non‐threatened species, and threatened species), we combined the ensemble of each group of species into a final ensemble (Figure 8; Breiner et al., 2015, 2018). In order to select the optimal habitat of bird species which occupy varying home ranges in such a heterogeneous environment, we preferred to rely more on practical binary (proportion of presence cells) maps instead of probability maps (mean habitat suitability; Progin, 2018; Scherrer et al., 2019), as it is more complicated to compare the probability of model predictions with binary data (presence‐absence) than to compare two variables at the same scale (Guisan et al., 2017). For this reason, a threshold value of 10% (the predicted value above which the model includes 90% of the training locations) was applied in probability map (minimal predicted area, MPA90%; Engler, Guisan, & Rechsteiner, 2004; Razgour et al., 2011; Scherrer et al., 2019). We then stacked and summed individual probabilities across all species predictions to yield maps of species richness that highlight optimal habitats and hotspots for each group of species. The predicted suitability of an area for bird species or the richness maps shows where there the best chance to find species is and allow us to understand how each group of species is distributed in the study area and could be a useful tool for nature practitioners (Distler, Schuetz, Velásquez‐Tibatá, & Langham, 2015; Dubuis et al., 2011).

## 3 RESULTS

### 3.1 Univariate models—Scale selection

**FIGURE 5:**
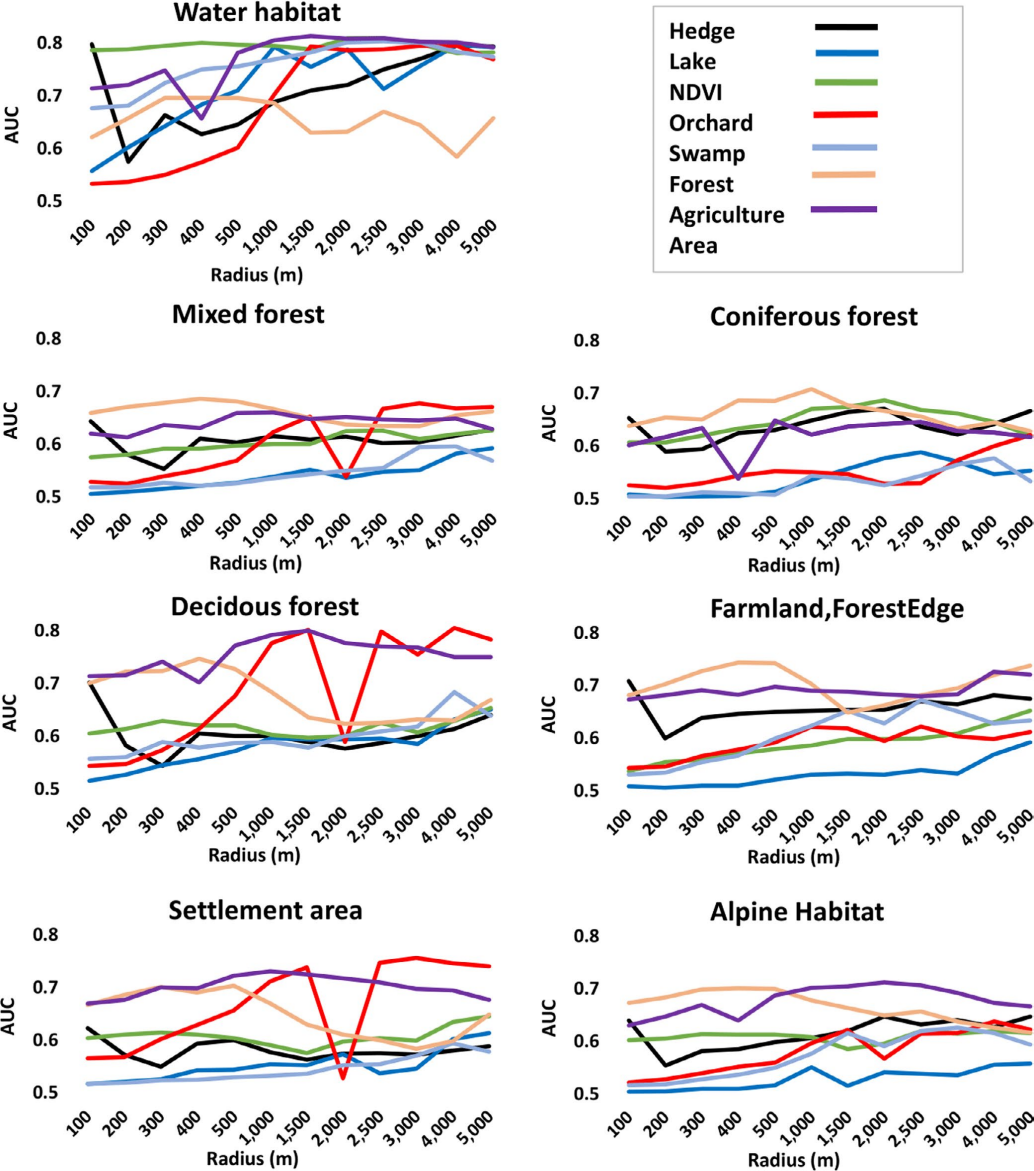
Performance of univariate models for each group of species in different habitats: Association (averaged area under the curve over the 50 runs of each univariate models) between the presence of each group of species and each focal variable at 12 spatial scales

The average predictive power of the focal variables is shown by AUC of univariate models for each focal variable across the scales (100–5,000 m) based on the test datasets of 7 species classes. Generally, our results showed that the primary predictors of species distribution were land use/cover variables measured at large scales with the neighboring window size ranging from 1,000 to 5,000 m (Figure 5), but with considerable variations among different species and variables. Bird species, in 7 different habitat, showed a high probability of presence/occurrence in areas located between 100 and 500 m (small scale) away from Hedge and Forest areas, while for Agriculture, Swamp, Orchard, Lake, and NDVI variables they had the highest probability of presence/occurrence across large scales (1,000–5,000 m; Figure 5). This (univariate model) approach could improve the selection of the best scale for each focal variable used in the following stage in the final ESMs.

### 3.2 ESMs—Model performance

**FIGURE 6:**
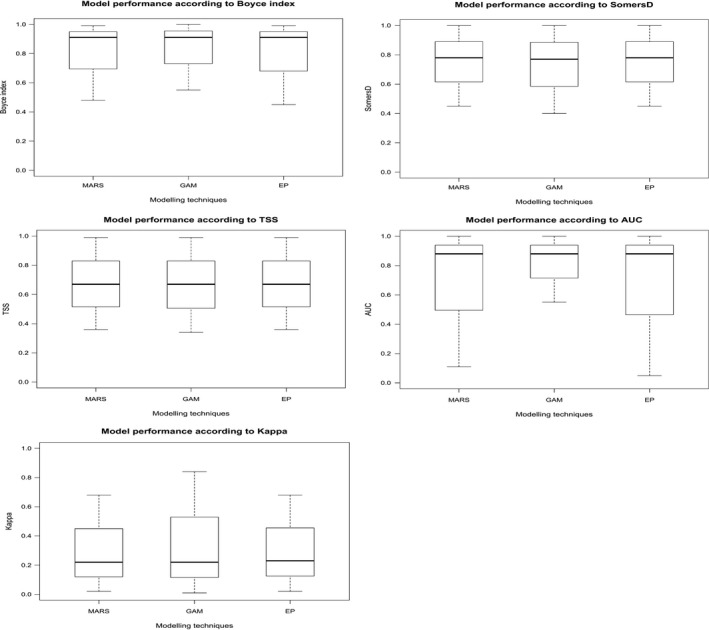
Evaluation of the ensemble of small models (ESMs). Boxplot of the Continuous Boyce Index, Somers'D, true skills statistic (TSS), area under the curve (AUC) and kappa, calculated for each run of the ESMs

The results have shown that both MARS and GAM modelling techniques have been performing very similarly and consistently based on all evaluation metrics (Boyce Index, SomernD, TSS, AUC and Kappa), there have been no significant improvement in performance of modeling techniques used within ESM framework (Scherrer et al., 2019). For instance, the "SomersD" index showed no significant differences in the performance of the ensemble prediction of models (EP) for all species, as the mean SomersD for MARS and GAM was 0.78 (±0.12 *SE*) and 0.77 (±0.13 *SE*) respectively. The mean TSS (MARS, GAM) and EP were similar for each modelling technique, equal to 0.67 (±0.15 *SE*), and it is true for other evaluation methods like AUC and Kappa (Figure 6). In summary, the performance of ensemble prediction, measured by the Continuous Boyce Index as one of the most reliable presence‐only evaluation metrics (Hirzel et al., 2006), has shown a good prediction accuracy for GAM models, ranging from 0.55 to 1 (*M* = 0.92, ±0.08 *SE*), and also for MARS models, ranging from 0.48 to 0.99 (*M* = 0.91, ±0.08 *SE*). All can be considered as very good models according to Araújo et al. (2005).

### 3.3 Variable importance in the final model (ESMs)

**FIGURE 7:**
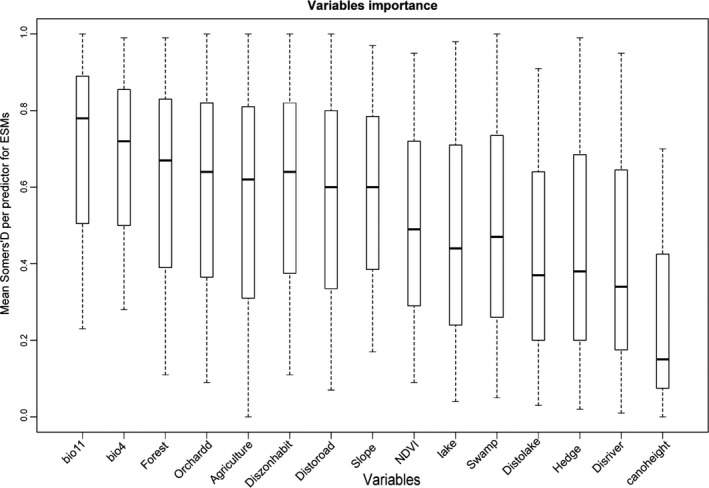
Variables contribution to three groups of the species. Boxplot of the variables contribution of each environmental variable among the 10 sets of pseudo absences. The value represents the proportion of the weights of the ensemble, which include the variable of interest

Figure 7, shows the contribution of each variable (see Table 1 for its acronyms and descriptions) to all 91 modeled species. In general, the patterns of habitat suitability highlighted by the ESMs are strongly linked to bioclimatic variables. For example, the most important relative contribution to model (0.79 and 0.72, respectively) to the average of all 91‐bird species was the Bio 11 (Mean Temperature of Coldest Quarter) and the Bio 4 (Temperature Seasonality), while for the focal variables, Forest (0.66), Orchard (0.64) and Agricultural (0.62) contributed a significant, but slightly lower, total weight of 91 modeled species. The third in total value assessments were Euclidean variables distance to residential area (Diszonehabit; 0.61) and distance to road (Distoroad; 0.58), and then Slope (0.60; the only topographic variable). The focal predictors, NDVI (0.49), Lake (0.46), Swamp (0.41) selected by univariate models, and the distance variable "Distolake" (0.45) were of less importance than the others. Eventually, there were the variables of poor performance, including Hedge (0.39), "Disriver" (distance to river; 0.35) and "canoheight" (canopy height mean; 0.16; Figure 7). Results of three different groups of bird species are provided in Figure S1.

### 3.4 ESMs—Species distribution maps

**FIGURE 8:**
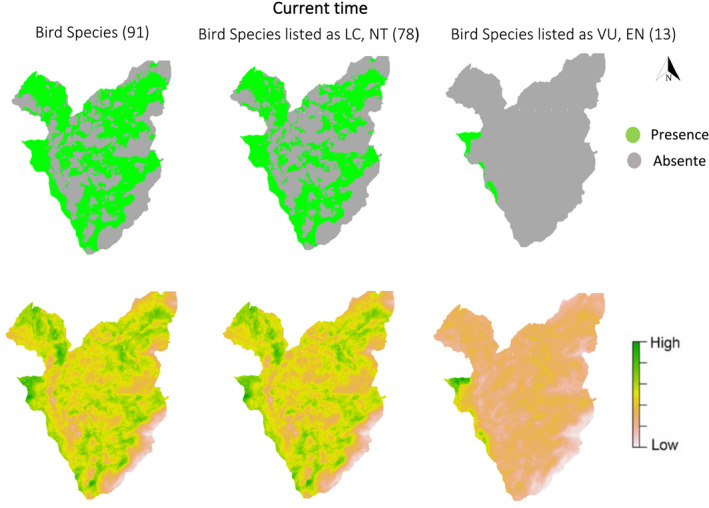
Prediction of the models for three groups of species. () Binary prediction of the ensemble of small models (ESMs) (upper row). () The final prediction of the ESMs (lower row)

In general, species richness predictions show that the areas close to coniferous forest, agricultural areas, and zone habitats (residential areas) of the highlands are the most suitable for 78 of species listed as LC, NT in the study area. Species richness maps of the 91 bird species and LC or NT in the national red list showed a pattern of moderate species richness distributed across the study area with higher habitat suitability in the eastern, central and northern parts of the study area near the highland area. However, this pattern varies, when 13 endangered species (VU or EN) are taken into consideration, which appear to occur only in lower slopes in the western part of the study area, near the deciduous forest, lake, and wetland (Figure 8).

## 4 DISCUSSION

This study supports the role of the best scale of effects of focal predictors (land cover and land use) at local scale (topography and micro‐climate) and the exclusion of poor focal variables (Bellamy et al., 2013; Progin, 2018) in bird species distribution in a complex landscape. This systematic approach to variable selection (univariate models with focal variables) and assessment of landscape's neighboring influence increased the power of the model predictions in our heterogeneous study area and our findings confirmed that bird species respond to ecological parameters at particular spatial scale (Graf et al., 2005; Rocchia, Luppi, Dondina, Orioli, & Bani, 2018). The additional important finding is that ESMs approach is highly efficient for modelling species distributions with limited number of species occurrences. ESMs, with a limited number of presence data and a large number of environmental variables, can be effectively used for modelling bird species, the generated models and distribution maps can be used as comprehensive and practical tools in biodiversity conservation decisions (Chamberlain, Pedrini, Brambilla, Rolando, & Girardello, 2016; Scherrer et al., 2019). This innovative study improved our knowledge and understanding of bird species niche variation, ecological priorities and distribution in the western Swiss Alps. It also supports practitioners to interpret the findings for identifying the environmental niche of the bird species (Guisan et al., 2017) and taking practical management decisions.

### 4.1 Univariate models

The occurrence of species, predicted by focal variables, significantly changed across all species and the relative importance of focal variables frequently changed with the radius and, in general, most were associated with focal variables measured at large scales between 1,000 and 5,000 m. Focal variables in both univariate and multivariate models (final models) of the majority of species, Hedge and Forest have generally been the most influential predictors of habitat suitability at the small scale (100–500 m; within foraging, nesting, and breeding range) in different habitats. It is remarkable that different group of species (7 groups) have similarly been identified in the small radius (100–500 m) of two focal variables Hedge and Forest. It could be interpreted that, among the 91 bird species included in our research, the majority are passerine birds (66 out of 91 species) which occupy agriculture habitats, and ecologically, breed, feed, and forage in forest edge areas that are rich in small trees and shrubs (Bauer, Bezzel, & Fiedler, 2012; Ludwig et al., 2012; Santangeli et al., 2018; Schaub, Kéry, Birrer, Rudin, & Jenni, 2011; Schwarz, Trautner, & Fartmann, 2018). Small‐scale habitats of two variables Hedge and Forest show the special feeding behaviors of species within the range of their nesting and feeding habitats that, for passerine in general, is a distance of around 300 m (Graf et al., 2005; Krištín & Kaňuch, 2017; Menz, Mosimann‐Kampe, & Arlettaz, 2009). It could be assumed that these 7 groups of species benefit from two variables (forest and hedge) as foraging sources of insect prey that coincide with nesting and feeding in small distances within the home range (Monsarrat et al., 2013). We may infer that, for two variables (Hedge and Forest), habitat suitability of the seven groups of species declines over larger scales (Roth, Amrhein, Peter, & Weber, 2008) and these variables are undesirable or may be disrupted by human activities at larger scales (Graf et al., 2005).

The presence/occurrence probability of majority of species in different habitats increased with increasing the radius of focal variables, “Agriculture area”, “Orchard”, “Lake”, “NDVI”, and “Swamp”, indicating that the strongest interaction with these focal variables was typically observed at larger scales (1,000–5,000 m). In these radius sizes, a greater number of species (Figure 5) benefit from these focal variables over larger scales, perhaps because these focal variables are faced with disruption such as urban and agricultural developments, transport growth, loss of connectivity (fragmentation) and loss of habitat at small scales in which bird species are threatened. Bird species are therefore forced to occupy suitable large‐scale habitats of these focal variables (Graf et al., 2005; Grüebler, Schuler, Spaar, & Naef‐Daenzer, 2015). We can conclude that bird species, in this mountainous area, are disturbed by small‐scale anthropogenic habitat disturbance for these five focal variables and it is not surprising that bird species in different habitats prefer these focal predictors at larger scales (Forman, Reineking, & Hersperger, 2002; Grüebler et al., 2015; Marzluff, 2001).

### 4.2 Species distribution maps

Creating species richness maps by summing species habitat suitability maps (HSMs) probably overestimates species richness because it does not account for limitation factors such as competition and dispersal that can affect species distribution (Bellamy et al., 2013; Graham & Hijmans, 2006). Prediction of habitat suitability in the highland areas (north to east of the study area) was generally higher and these highland areas support higher diversity of species (all species, threatened and non‐threatened species) except for VU and EN species. The richness maps of the threatened species signify a different pattern from those of the non‐threatened species and the main distribution area of this group of birds is only the western part of the study area located below 1,000 m that can be regarded as the area near the water habitats such as Lake Geneva and wetlands with well‐vegetated banks and parts of the deciduous forest that have been used as foraging and feeding sites. Lowland areas can be considered suitable for the VU and EN species due to the warmer climate and proximity to water habitats like lake and wetlands in the study area. Generally, climatic and focal variables “Orchard” and “Forest” play an important role in the distribution of species (Figure 7) and most species seem to respond negatively to global warming as they are forced to shift to the highland area (Maggini et al., 2011, 2014).

In some parts of lowland areas and the western quadrant of the study area, prediction maps of non‐threatened species (mostly passerine bird species in agricultural habitat) were also higher, which can be interpreted as the importance of arable land and wide‐leaved forest for feeding and nesting of this group of species. These areas are richer in resources of foraging and nesting. The findings of these study thus emphasize the importance of forests for the survival of bird species contributing to biodiversity conservation. The findings suggest that, despite the high anthropogenic effects in the field, most of the species have a strong relationship with the forest area. Conservation efforts should therefore concentrate on forest area management and conservation in order to optimize habitat for these species.

Our research allows us to claim that forest regions are the most potential areas for feeding and nesting of these species (Brüngger & Estoppey, 2008; Carpenè et al., 2006). According to our analysis, we are also able to conclude that lower temperatures play a major role in the distribution of most species in this mountainous area, and that these species react negatively to higher temperatures as some are predicted to shift and some already shifted to higher altitudes according to some case studies in Switzerland (see Maggini et al., 2011, 2014), which indicates that bird species are likely affected in breeding season by climate change due to increasing temperatures in the breeding season. As the available habitat suitability is reduced, the distributional movements can thus be a common response to this situation (Revermann et al., 2012). Our findings shows that climate in the western Swiss Alps, particularly temperature, is the main driving factor for birds and during breeding seasons, minor changes in temperature can push species up. Our results have proved that the bioclimatic variables have the most important effects on species distribution and the important part has been played by "Bio11" and "Bio 4" in shaping the distribution of these species.

## 5 MAIN CONCLUSIONS

One first shortcoming in this study was the limitations of selecting maximum scales in univariate models due to the heterogeneity and size of the study area and similar responses of some species for many variables. Some species, for example, have different ecological behavior and strategies for feeding, nesting and long‐distance flight and probably prefer scales of more than 5,000 m. We eventually selected 5,000 m as a maximum scale for all focal variables to address the problem of the size of the study area. The multi‐scale approach that incorporates focal variables with the strength of ESMs was efficient in identifying the complexities of bird localization as species of high mobility in a heterogeneous ecosystem and proved to outperform single scale modelling. This study  reveals the significance of lower and seasonal temperatures at higher elevations, as well as forest and farmland edge, for the bird species in the study area. The availability of data for certain species, due to difficulty of detection, rare and threatened status, was also limited in this research, but prediction advantages of ESMs enabled the models to consider poorly sampled bird species and model the distribution of species with limited availability of occurrence data. ESM is a powerful strategy to understand the ecology of complex and heterogeneous environments, and nature reserve managers may make better use of the models to estimate the effects of climate change and land use, such as agriculture utilization and urbanization, on target species. Distribution maps of the area under study will help to identify the locations of the most threatened and endangered bird species biodiversity and improve conservation efforts toward the identification of bird hotspot. In this study, we only tested the framework with bird species, but this strategy could be applied to other taxa and here we encourage future studies to also examine this modelling approach particularly in case of limited presence data and rare species.

## CONFLICT OF INTEREST

None declared.

## AUTHOR CONTRIBUTION

**Nasrin Amini Tehrani:** Conceptualization (lead); data curation (lead); formal analysis (equal); funding acquisition (lead); investigation (equal); methodology (equal); project administration (equal); resources (equal); software (equal); visualization (equal); writing – original draft (lead); writing – review and editing (equal). **Babek Naimi:** Conceptualization (supporting); data curation (supporting); formal analysis (equal); methodology (equal); project administration (supporting); resources (equal); software (supporting); supervision (equal); validation (supporting); visualization (supporting); writing – original draft (supporting); writing – review and editing (supporting). **Michel Jaboyedoff:** Conceptualization (supporting); data curation (supporting); formal analysis (equal); funding acquisition (supporting); investigation (supporting); methodology (equal); project administration (equal); resources (equal); software (supporting); supervision (lead); validation (lead); visualization (supporting); writing – original draft (supporting); writing‐review and editing (supporting).

## Supporting information

Supplementary Material

## Data Availability

The data that support the findings of this study are available from the Swiss Ornithological Institute but restrictions apply to the availability of these data, which were used under license for the current study, and so are not publicly available.
