# Supplementary material for: Toward community predictions: Multi‐scale modelling of mountain breeding birds' habitat suitability, landscape preferences, and environmental drivers
Source: Ecol Evol. 2020 Apr 29;10(12):5544–57. doi: 10.1002/ece3.6295 (PMC7319251; doi:10.1002/ece3.6295)
Supplement: Supplementary file 1 — Supplementary Material [file ECE3-10-5544-s001.docx]

Appendix S1 Details on environmental predictors

“**Temperature [°C]**

The temperature data are derived from daily MeteoSwiss Grid-Data Products at 1 km resolution for 1981-2010. Maps were downscaled from 1 km to 25 m using local linear regressions with elevation T1km = a1km + b1km * Elevation1km in a moving window of 5 km radius. Intercepts a1km and slopes b1km are then disaggregated at 25 m and smoothed spatially with conic density. T25m = a25m + b25m * Elevation25m is then applied. (Broennimann, comm. pers.). The minimal temperatures were averaged daily between 1981 and 2010, and temperature from May to August were averaged. Final temperature map was aggregate to 100 m resolution by mean.”

“**Slope [°]**

Slope was calculated from a digital terrain model (DTM) at a 25 m resolution (Swisstopo OFT) by a moving window. Slope at 25 m was furtherly aggregated to 100 m resolution by mean.”

“**Distance to zonhabitats – Diszonhabitat [m]**

The Euclidean distance to buildings was calculated in ArcGIS 10.2 (www.esri.com) with the near tool and a layer of every building of the area (Vector25 2008; Swisstopo OFT). The Euclidean distance is calculated from the center of each of the cells from a 100 m resolution raster to the nearest building.”

“**Distance to river– Disriver [m]**


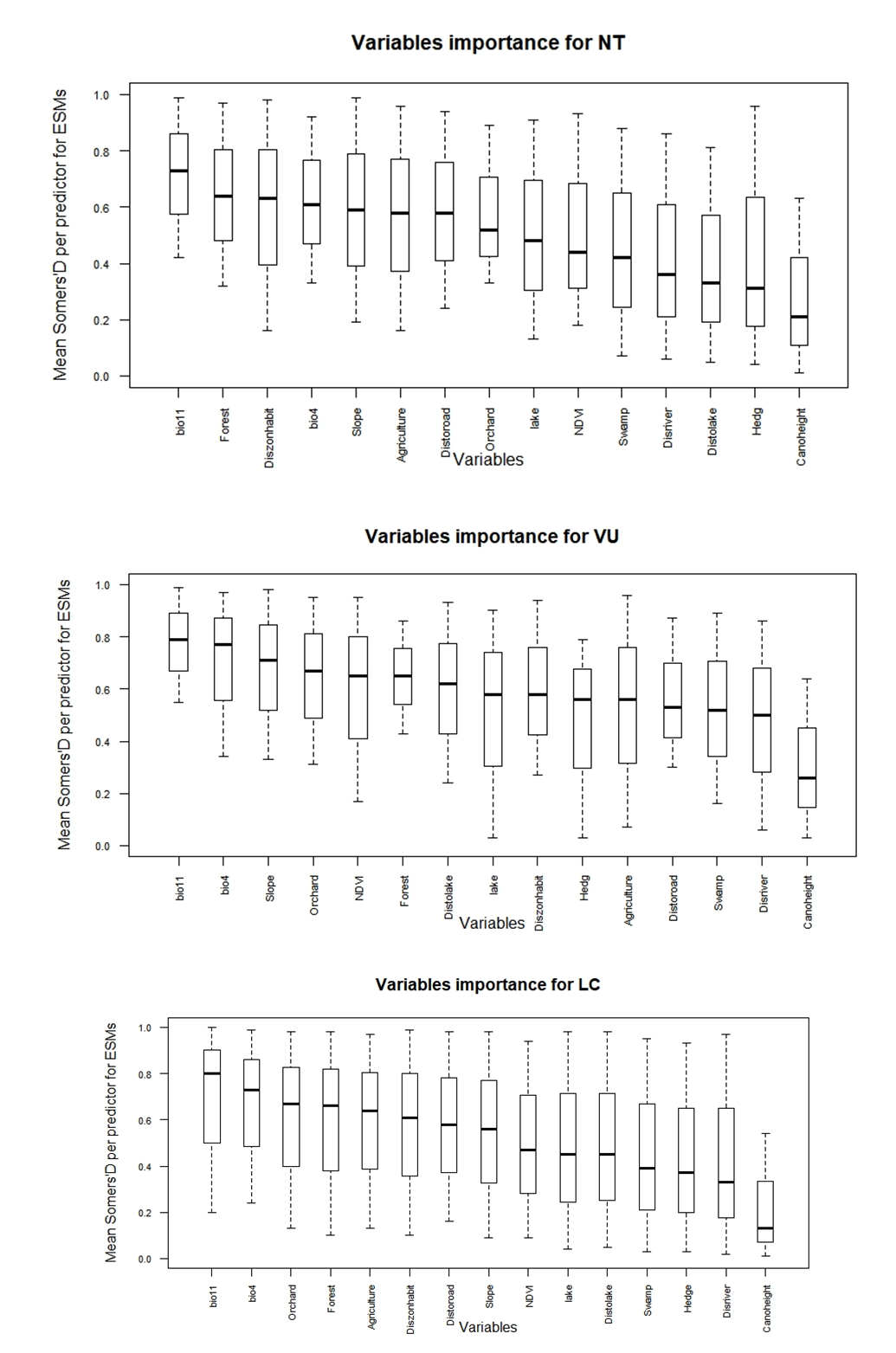
The Euclidean distance to rivers was calculated in ArcGIS 10.2 (www.esri.com) with the near tool and a layer of forests of the area (Vector25 2008; Swisstopo OFT). The Euclidean distance is calculated from the center of each of the cells from a 100 m resolution raster to the nearest forest.”

“**Distance to roads – Distoroads [m]**

The Euclidean distance to roads was calculated in ArcGIS 10.2 (www.esri.com) with the near tool and a layer of every road of the area (Vector25 2008; Swisstopo OFT). The road layer does not distinguish between road size and type, every crossable road. The Euclidean distance is calculated from the center of each of the cells from a 100 m resolution raster to the nearest road.”

“**Distance to lake – Distolake [m]**

The Euclidean distance to water was calculated in ArcGIS 10.2 (www.esri.com) with the near tool and a layer of watercourses and lakes of the area (Vector25 2008; Swisstopo OFT). The watercourse layer encompasses all kind of watercourses from small stream to big river. The Euclidean distance is calculated from the center of each of the cells from a 100 m resolution raster to the nearest source of water, water either course or lake.”

“**Variance of canopy heights – canoheight [m2]**

The canopy heights were calculated by doing the difference of the digital terrain model (DTM) to the digital surface model (DSM) at 1 m resolution (Swisstopo OFT) and this difference was furtherly masked with a forest layer of the area. Final canopy map was aggregate to 100 m resolution by variance to have a heterogeneity index of the canopy heights.”

“**Normalized difference vegetation index – NDVI [-1; 1]**

Normalized difference vegetation index, calculated with an image of the area at 10 m resolution rescaled to 100 m by mean in ArcGIS 10.2 (www.esri.com). The images were taken in September and were provided by Swisstopo, OFT. A mean was calculated with a moving window of different radius to create ndvi_focal.”

“**Agriculture. Area – Agriculture [%]**

The proportion of agriculture area over the area calculated with a land-cover layer (Geostat 2013/2018, OFS) reclassified into two classes (Agriculture. Area or not). The proportion is calculated with a moving window of different radius to produce the proportion of the land-cover available in the surroundings of each cell.”

“**Forest area – Forest [%]**

The proportion of forest area over the area calculated with a land-cover layer (Geostat 2013/2018, OFS) reclassified into two classes (forest area or not). The proportion is calculated with a moving window of different radius to produce a proportion of the land-cover available in the surroundings of each cell.”

“**Hedge – Hedge [%]**

The proportion of hedge (Geostat 2013/2018, OFS) reclassified into two classes (Hedge or not). The proportion is calculated with a moving window of different radius to produce the proportion of the land-cover available in the surroundings of each cell.”

“**Lake—lake [%]**

Proportion of cells including either a lake (Vector25 2008; Swisstopo OFT) reclassified into two classes (lake or not). The proportion is calculated with a moving window of different radius to produce proportion of the land-cover available in the surroundings of each cell.”

“**Orchard– Orchard [%]**

The proportion of orchard over the area calculated with a land-cover layer (Geostat 2013/2018, OFS) reclassified into two classes (Orchard or not). The proportion is calculated with a moving window of different radius to produce the proportion of the land-cover available in the surroundings of each cell.”

“**Swamp – Swamp [%]**

Proportion of swamp over the area calculated with a land-cover layer (Geostat 2013/2018, OFS) reclassified into two classes (swamp and or not). The proportion is calculated with a moving window of different radius to produce proportion of the land-cover available in the surroundings of each cell.” (from Progin et al., 2018; Scherrer et al., 2019)

**Table S 1:** Autocorrelation of the environmental variables


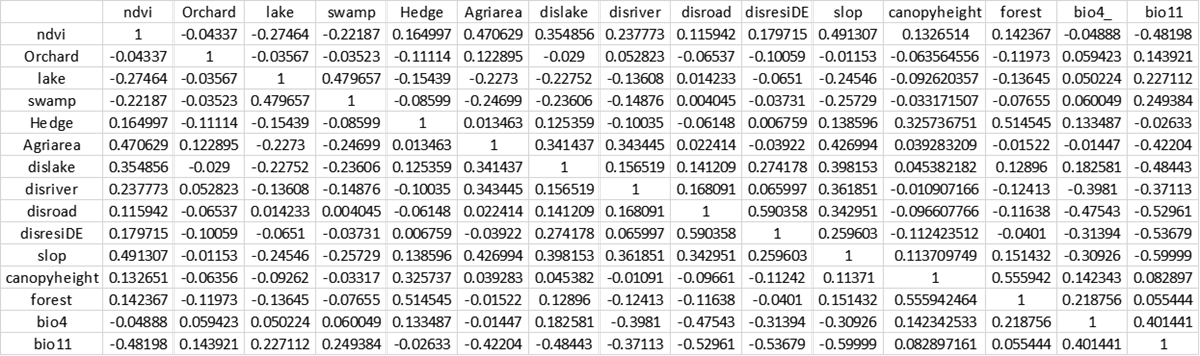


**Table S1**: The selected variables were not too highly correlated (spearman correlation (> 0.7); Dormann et al., 2013). To reduce collinearity, we tested correlations for all predictors and only one was taken if two of them were found to be highly correlated (r. 0.7). The decision of which to include was based on ecological relevance (Gogol-Prokurat, M. 2011). For details on the environmental variables, see Table 1 and supplementary methods.


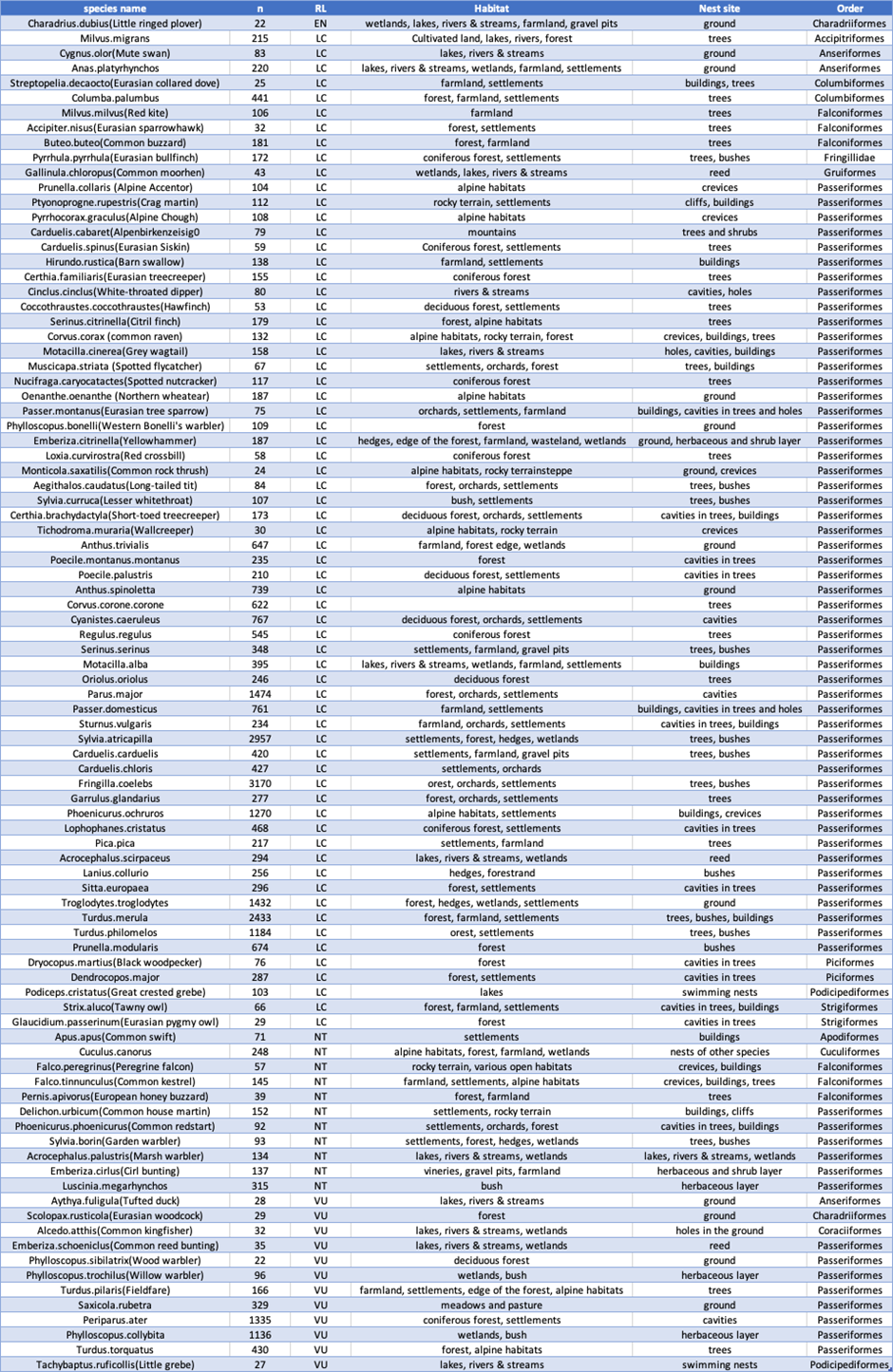


**Table S2:** Complete species list with information about the number of occurrences (n), the Red List status (RL), endangered (EN), vulnerable (VU), near threatened (NT) and least concern (LC).


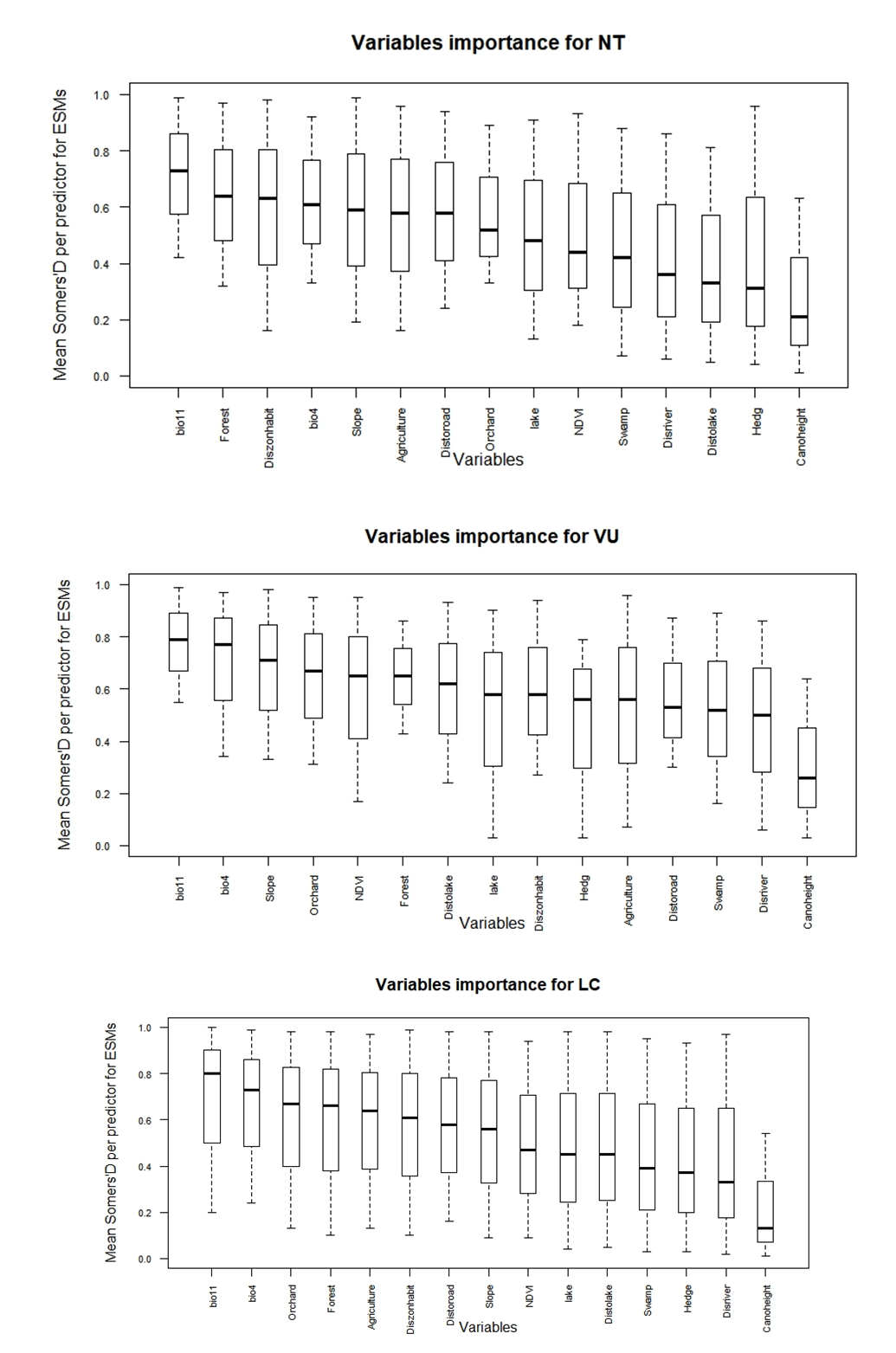


**Figure S2:** Topography map of the study area

**Figure S1:** Variables contribution for three groups of the species. Boxplot of the variables contribution of each environmental variable among the 10 sets of pseudo absences. The value represents the proportion of the weights of the ensemble, which include the variable of interest


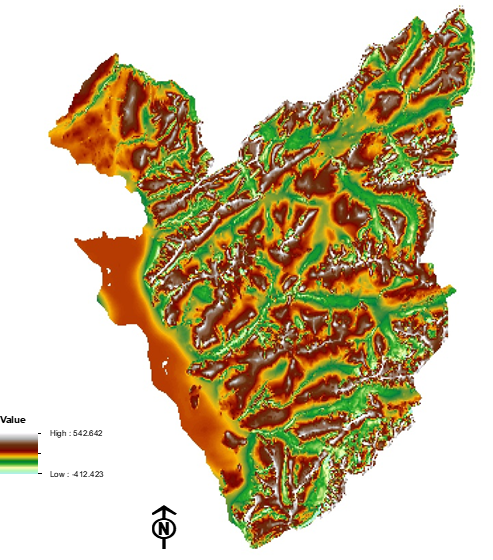


**Figure S2**: Topography map of the study area

**Figure S3:** Spatial correlation of model residuals


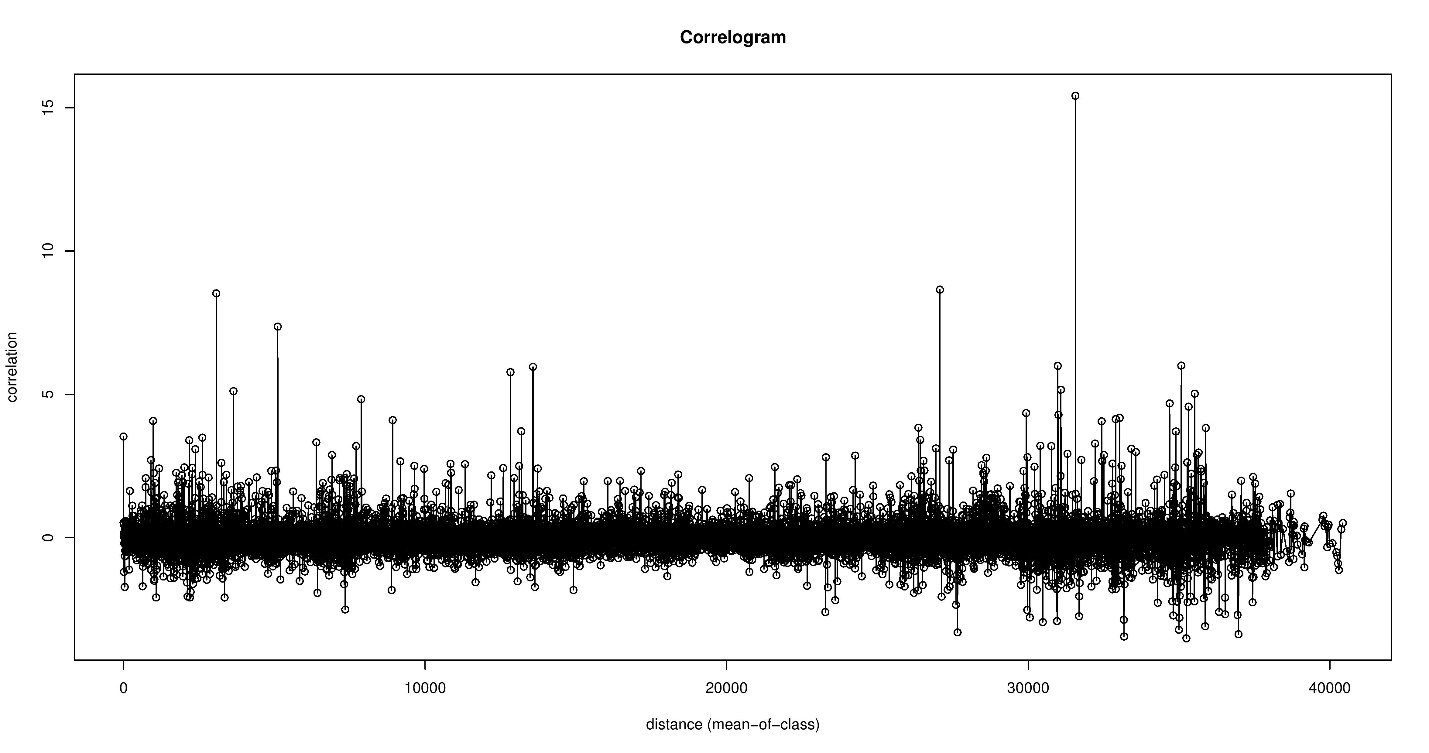


**Figure S3**: For detecting spatial autocorrelation in the residuals, Moran’s I test was done. We found that p-value for testing spatial autocorrelation is significant (p < 0.05). We, therefore, plot spatial correlation against distances between the occurrences of the species. The distances were evaluated by using a Mantel correlogram (Gusian et al, 2017).

**References**

Gogol-Prokurat, M. (2011). Predicting habitat suitability for rare plants at local spatial scales using a species distribution model. Ecological Applications, 21(1), 33-47.

Guisan, A., Thuiller, W., & Zimmermann, N. E. (2017). Habitat suitability and distribution models: with applications in R. Cambridge University Press.

Progin, D. (2018) Modelling habitat suitability of bats in the Prealps (Master thesis), Lausanne University, Lausanne, Switzerland.

Scherrer, D., Christe, P., & Guisan, A. (2019). Modelling bat distributions and diversity in a mountain landscape using focal predictors in ensemble of small models. Diversity and Distributions. https://doi.org/10.1111/ddi.12893
